# A hybrid spatiotemporal deep belief network and sparse representation-based framework reveals multilevel core functional components in decoding multitask fMRI signals

**DOI:** 10.1162/netn_a_00334

**Published:** 2023-12-22

**Authors:** Limei Song, Yudan Ren, Shuhan Xu, Yuqing Hou, Xiaowei He

**Affiliations:** School of Information Science and Technology, Northwest University, Xi’an, China

**Keywords:** Multitask classification, Task-based fMRI, Deep belief network, Sparse representation, Functional brain network

## Abstract

Decoding human brain activity on various task-based functional brain imaging data is of great significance for uncovering the functioning mechanism of the human mind. Currently, most feature extraction model-based methods for brain state decoding are shallow machine learning models, which may struggle to capture complex and precise spatiotemporal patterns of brain activity from the highly noisy fMRI raw data. Moreover, although decoding models based on deep learning methods benefit from their multilayer structure that could extract spatiotemporal features at multiscale, the relatively large populations of fMRI datasets are indispensable, and the explainability of their results is elusive. To address the above problems, we proposed a computational framework based on hybrid spatiotemporal deep belief network and sparse representations to differentiate multitask fMRI (tfMRI) signals. Using a relatively small cohort of tfMRI data as a test bed, our framework can achieve an average classification accuracy of 97.86% and define the multilevel temporal and spatial patterns of multiple cognitive tasks. Intriguingly, our model can characterize the key components for differentiating the multitask fMRI signals. Overall, the proposed framework can identify the interpretable and discriminative fMRI composition patterns at multiple scales, offering an effective methodology for basic neuroscience and clinical research with relatively small cohorts.

## Author Summary

Decoding different cognitive processes using task-based functional magnetic resonance imaging (tfMRI) is crucial for understanding the relationship between brain activities and cognitive states. However, existing machine learning–based feature extraction methods for decoding brain states may struggle to capture the complex and precise spatiotemporal patterns of brain activity from the highly noisy raw fMRI data. Additionally, current deep learning–based end-to-end decoding models struggle to unveil interpretable components in tfMRI signal decoding. To address these limitations, we proposed a novel framework, the hybrid spatiotemporal deep belief network and sparse representations (DBN-SR) framework, which effectively distinguished multitask fMRI signals with an average accuracy of 97.86%. Furthermore, it simultaneously identified multilevel temporal and spatial patterns of multiple cognitive tasks. By utilizing a novel ratio-of-activation metric, our framework unveiled interpretable components with greater classification capacity, offering an effective methodology for basic neuroscience and clinical research.

## INTRODUCTION

For years, researchers have been attempting to decode the human brain states based on functional magnetic resonance imaging (fMRI) data (Dehaene et al., 1998; Haynes & Rees, 2006; Jang, Plis, Calhoun, & Lee, 2017; Rubin et al., 2017), where distinguishing different cognitive tasks from fMRI data and extracting discriminative fMRI composition patterns are effective means to improve our understanding of the relationship among current cognitive tasks, brain responses, and individual behavior (Friston, 2009; Logothetis, 2008). To decode meaningful neurological patterns embedded in diverse task-based fMRI data, various computational and statistical methods have been proposed in the last decades. The most widely used brain state decoding strategy is multivoxel pattern analysis (MVPA) (Davatzikos et al., 2005; Jang et al., 2017; Kriegeskorte & Bandettini, 2007). Despite its popularity, its commonly used classification strategy support vector machine (SVM) usually struggles to perform well on high-dimensional fMRI data and thus requires effective techniques for feature selection/extraction (LeCun, Bengio, & Hinton, 2015; Vieira, Pinaya, & Mechelli, 2017). Hence, the feasibility of feature selection/extraction has been investigated using various machine learning methods (LeCun et al., 2015; Vieira et al., 2017; Zhang et al., 2016). However, most of these machine learning methods rely on shallow models, and their shallow nature may hinder them from effectively capturing nonlinear relationships in the highly noisy fMRI raw data, resulting in difficulties in extracting complex and specific spatiotemporal features (Qiang et al., 2020; Rashid, Singh, & Goyal, 2020; Varoquaux & Thirion, 2014).

Recently, studies applying deep learning models such as deep neural network (DNN) and convolutional neural networks (CNN) to decode brain states based on task-based fMRI signals have been reported (Hu et al., 2019; Liu, He, Chen, & Gao, 2019; Koyamada et al., 2015; Zhang, Tetrel, Thirion, & Bellec, 2021). Such deep learning models take the advantage of being a multilayer architecture by stacking multiple building blocks with similar structure, which has demonstrated the ability to significantly reduce noises in raw fMRI data and model the nonlinear relationships among neural activities of brain regions, allowing for the extraction of multilevel spatiotemporal features (Bengio, Courville, & Vincent, 2012; Najafabadi et al., 2015; Ren, Xu, Tao, Song, & He, 2021). Nevertheless, there are still some limitations in current brain state decoding strategies based on deep learning models. First, as large-size samples are indispensable for the deep learning model, current decoding models are not suitable for small datasets (Liu et al., 2017b; Litjens et al., 2017; Wang et al., 2020; Wen et al., 2018). For example, Wang et al. (2020) proposed a DNN-based model for tfMRI signal classification, which requires 1,034 subjects, making it less practical for clinical populations. Second, most of the decoding models based on deep learning are end-to-end learning and the explainability of such models is elusive (Hu et al., 2019; LeCun et al., 2015; Wang et al., 2020). Recently, some researchers have attempted to define the key components for decoding brain states using the machine learning method. For example, our previous study based on sparse dictionary learning has determined that the key components for multitask classification tend to be functional brain networks (FBNs) (Song, Ren, Hou, He, & Liu, 2022). Another research has shown that artifact components such as movement-related artifacts are significantly more informative with respect to the classification accuracy of the multitask electroencephalogram (EEG) signals (McDermott et al., 2022). However, uncovering the interpretable key features in decoding tfMRI signals has received much less attention.

Due to the pitfalls in existing research, it is desirable to develop an appropriate framework capable of identifying the interpretable and discriminative fMRI composition patterns embedded in multitask fMRI data. Thus, in this study, we aim to extract both multilevel group-wise temporal features and spatial features from tfMRI signals, and define interpretable classification features for multitask fMRI data simultaneously. Recent studies have revealed that the deep belief network (DBN) can effectively identify multilayer spatial and temporal features from fMRI signals (Dong et al., 2020; Ren et al., 2021), which is typically stacked by multiple Boltzmann machine (Hinton & Sejnowski, 1986) and thus can naturally act as a multilevel feature extractor. Furthermore, these prior studies have integrated the least absolute shrinkage and selection operator (LASSO) regression with the DBN model, indicating the efficacy of LASSO regression in extracting relevant spatial patterns. Thus, we here proposed a novel two-stage feature extraction framework based on hybrid DBN and sparse representations framework (DBN-SR) to decode multitask fMRI signals with the capability of extracting multiscale deep features. Specifically, the DBN model was utilized to capture multilevel group-wise temporal features, based on which the individual spatial features were estimated by LASSO regression. Subsequently, a sparse representation method that combines dictionary learning and LASSO regression was utilized to further characterize the group-wise spatial features and individual spatiotemporal features for the purpose of classification. Based on the correspondence between the individual classification features and the group-wise spatial features, a relationship between the decoding capability of classification features and their spatial patterns can be effectively established, which can facilitate the interpretation of neural implications associated with the classification features. Finally, due to its strong generalization capabilities in small sample sizes, SVM was employed for the multiclass classification task.

Our results demonstrated that the proposed framework could successfully classify seven task fMRI signals on a relatively small dataset. Moreover, by taking advantage of DBN in extracting mid-level and high-level features and sparse coding in brain functional network representation (Lv et al., 2015a; Ren et al., 2021; Song et al., 2022), our framework could effectively characterize the multilevel spatiotemporal features embedded in multitask fMRI signals, which provides the bases to identify the interpretable key components for well characterizing and differentiating multitask signals. Overall, the proposed model can disclose the underlying neural implications of key components with greater classification capacity, offering an effective and interpretable methodology for decoding fMRI data.

## MATERIALS AND METHODS

### Overview

**Figure 1.**
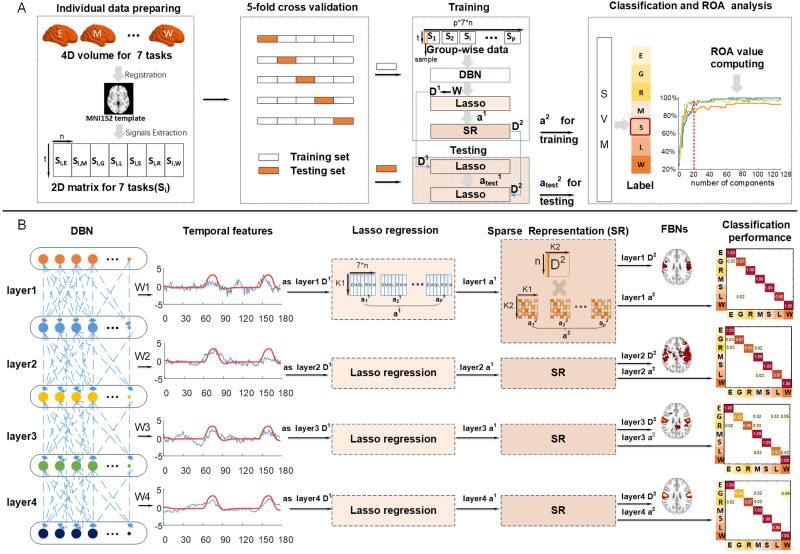
The overview of hybrid deep belief network and sparse representation framework (DBN-SR). (A) The pipeline of multitask fMRI data classification analysis via the proposed model. The seven capital letters refer to seven different tasks, respectively (E: emotion, G: gambling, R: relational, M: motor, L: language, S: social, and W: work memory). (B) The detailed illustration of using DBN and SR model to extract multilevel temporal features, spatial features, and features for classification from multitask fMRI signals. In the second block, the blue line represents temporal features derived from the weights of DBN, while the red line represents task design paradigms.

The framework of our proposed method is illustrated in Figure 1. The pipeline of the proposed framework can divide into four stages: (1) individual data preparation, (2) data preparation for fivefold cross-validation, (3) training and testing process, and (4) SVM-based classification and ratio-of-activation (ROA) analysis (Figure 1A). In the data preparation stage, each individual’s tfMRI data of seven different tasks were extracted and then spatially concatenated to one signal matrix (the first panel in Figure 1A). In this work, fivefold cross-validation was performed for model validation, thus the whole dataset was randomly divided into five folds (the second panel in Figure 1A). In training process, four folds were served as training set, and the tfMRI signal matrices of all the subjects in training set were spatially concatenated to a multisubject signal matrix. Then, the DBN model was applied to training set to derive the weight matrix W, which served as group-wise temporal features ***D***^1^. Then, the LASSO regression aims to extract the corresponding loading coefficient *a*^1^ based on the defined temporal dictionary ***D***^1^. In the second stage of our model, the loading coefficient ***α***^1^ was employed as input to sparse representations (SR) model, where they were decomposed into group-wise dictionaries ***D***^2^ and loading coefficient ***α***^2^. In testing process, the individual signal matrix in testing set and the group-wise dictionary ***D***^1^ obtained during the training phase was utilized as the inputs to the LASSO regression. This yielded the loading coefficients $\alpha_{\text{test}}^{1}$. Subsequently, employing $\alpha_{\text{test}}^{1}$ and the ***D***^2^ obtained during the training phase, we performed a second LASSO regression to obtain $\alpha_{\text{test}}^{2}$, which were then used as the classification features for the testing subjects (the third panel in Figure 1A). Note that during the training phase, we utilized the independent training data to learn and train regularization parameters employed for LASSO regression, as well as the group-wise dictionaries ***D***^1^ and ***D***^2^, without using any information from the test data. Afterward, to further assess the multitask fMRI data classification performance of proposed model, the loading coefficient ***α***^2^ derived from training set was used to train SVM for classification, where the loading coefficient $\alpha_{\text{test}}^{2}$ derived from the testing set was then fed into this trained SVM model to identify the testing set labels (the last panel in Figure 1A).

Our DBN-SR based framework can also identify the multilevel temporal features, spatial features, and features for multitask classification (Figure 1B). Specifically, the DBN model took fMRI time series from training data as input and produced a weight matrix W for each layer respectively, which represent the multilayer temporal features of group-wise tfMRI signals (the first two panels in Figure 1B). These multilayer temporal features W were served as the temporal dictionary ***D***^1^ and used as input to the LASSO algorithm to regress corresponding loading coefficient ***α***^1^, which represents individual-level spatial patterns (the third panel in Figure 1B). Next, the loading coefficient ***α***^1^ was used as the input of SR stage to derive the common dictionary ***D***^2^ and the loading coefficient ***α***^2^, which represent group-wise spatial patterns and features for multitask classification for each layer, respectively (the last three panels in Figure 1B).

### Data Acquisition and Preprocessing

We employed the seven-task fMRI data from Q1 release of Human Connectome Project (HCP) in this study (Barch et al., 2013). The details of tfMRI data acquisition and preprocessing pipeline could be referred to our previous study (Song et al., 2022).

Specifically, the seven tasks are emotion, gambling, relational, motor, language, social, and working memory (WM). The number of time points for each task is shown in Table 1. As the tfMRI data consist of different time points, we truncated all tfMRI signals to the same time length (176 frames). In this work, 60 subjects were used from the released dataset.

**Table 1.** Details of the condition and frames for seven tasks

Task	Emotion	Gambling	Relational	Motor	Language	Social	WM
Condition	2	2	2	6	2	2	8
Frames	176	253	232	284	316	274	405

The truncation preprocessing, unavoidably, influences the integrity of task design. For instance, four conditions are excluded from the WM task due to data truncation. Nonetheless, in terms of other tasks, the truncated tfMRI data include not less than one block for all events (Figure S1).

### Data Preparation

First, we extracted the whole-brain fMRI signal for each subject using the standard MNI152 template as the mask, resulting in each two-dimensional matrix. Then the signal matrices of the seven tasks for each subject were spatially concatenated into a large matrix $S_{i}^{1}(S_{i}^{1}=[S_{i,E}^{1},S_{i,G}^{1},S_{i,R}^{1},S_{i,M}^{1},S_{i,L}^{1},S_{i,S}^{1},S_{i,W}^{1}]\in R^{t\times \left(n\times 7\right)})$, where $S_{i,E}^{1}\in R^{t\times n}$ had *t* time points and *n* voxels. The seven capital letter subscripts refer to seven different tasks respectively (E: emotion, G: gambling, R: relational, M: motor, L: language, S: social, and W: work memory). TfMRI time series for each voxel were normalized to derive zero mean and unit norm. In this work, fivefold cross-validation scheme was chosen. Thus, 60 subjects were randomly divided into five equal folds. In each iteration, one fold (12 subjects) was taken for testing and the rest four (48 subjects) for training. It is noteworthy that the training and testing sets for each iteration were completely independent. Then, the multitask fMRI signal matrices of all the subjects in the training set were spatially concatenated to compose a multisubject fMRI matrix ***S***^1^ = $[S_{1}^{1},S_{2}^{1},\ldots ,S_{p}^{1}]\in R^{t\times \left(n\times 7\times p\right)}$, where *p* is the number of training subjects (*p* = 48) (Figure 1A).

As whole-brain fMRI data generally contain enormous voxels, the group-wise tfMRI signals consisting of multiple tasks and subjects exhibit relatively high dimensionality, inevitably resulting in an overloaded computational burden and memory consumption. To tackle these problems, we randomly selected only 10% of voxels’ whole-brain signals for each subject in training stage (Liu et al., 2017a; Song et al., 2022). To ensure the uniform distribution of sampled voxels across different brain regions, we employed the Fisher-Yates shuffle algorithm implemented by the “randperm” function in MATLAB, known for generating random permutations with a uniform distribution (Fisher & Yates, 1938). The distribution of the randomly selected 10% voxels across all subjects can be found in the Supporting Information (Figures S6 and S7).

### Deep Belief Network Model-Based Analysis

In this work, we chose DBN to extract group-wise temporal features based on previous research demonstrating its ability to identify meaningful FBNs (Qiang et al., 2020; Ren et al., 2021). In general, DBN can be regarded as stacked blocks of restricted Boltzmann machines (RBMs) (Hinton, Osindero, & Teh, 2006), an energy-based probability generation model that simulates the potential distribution of input data via interactions between visible and hidden variables. While units between visible layer *v* and hidden layer *h* are connected by weights, there is no connection within the layer. As a multiple stacked RBM model, the DBN model is designed to learn and train weights for each layer. As described in Fischer and Igel (2012) and Hu et al. (2018), the energy function of the DBN model adopted to update the weights layer by layer is defined as follows:

$$E(v,h)=\sum b_{i}v_{i}-\sum b_{j}h_{j}-\sum v_{j}h_{j}w_{j}$$ (1)

Where *v*_*i*_ and *h*_*j*_ refer to the activation state of two layers; *b*_*i*_ and *b*_*j*_ represent their bias; *w*_*j*_ indicate the weight between layer *i* and layer *j*.

As introduced in the previous section, the tfMRI signals of randomly selected 10% voxels in each individual’s whole brain of multitask in training set were spatially concatenated to generate a multisubject fMRI matrix for model training, and thus the group-wise tfMRI time series (176 time points) were taken as training samples for the DBN model. In our work, the neural architecture of DBN model was set as 4 layers and 128 neurons experimentally and empirically (see Parameter Selection section). Specifically, the number of visible variables *t* is the same as the number of time points of fMRI signal (i.e., 176 in our study), and the number of hidden variables *k*1 in each hidden layer represents the number of latent components expressed in fMRI data (*k*1 = 128). The DBN model was adopted to model group-wise tfMRI matrix ***S***^1^ to obtain a weight matrix *w*_*j*_ from each layer. The weight matrix of visible layer is represented by *w*_1_*ϵR*^*t*×*k*1^, and the weight matrix of each hidden layer refers to *w*_*j*_*ϵR*^*k*1×*k*1^ (*j* = 2, 3, 4). The multilayer temporal features *W*_*j*_ in each layer of DBN model can be derived by successive multiplication of the weight matrices on the adjacent layers (*W*_j_*ϵR*^*t*×*k*1^), that is, *W*_4_ = *w*_4_ * *w*_3_ * *w*_2_ * *w*_1_, *W*_3_ = *w*_3_ * *w*_2_ * *w*_1_, *W*_2_ = *w*_2_ * *w*_1_, *W*_1_ = *w*_1_. Since each sample input to the DBN model consists of all time points for each voxel, the weights *w*_*j*_ (*j* = 1, 2, 3, 4) across four layers represent the temporal features of the input fMRI data at different levels of abstraction. Thus, the successive multiplication of weight matrix *W*_*j*_ (*j* = 1, 2, 3, 4) obtained from each layer of the DBN model represents multilevel temporal features embedded in fMRI signals.

Drawing inspiration from the successful application of LASSO regression for deriving spatial features in previous studies (Haufe et al., 2014; Lee, Jeong, & Ye, 2013), we performed the LASSO regression to derive individual spatial features. Specifically, the multilayer temporal features *W*_*j*_ derived by the DBN model were normalized and then served as the temporal dictionary ***D***^1^*ϵR*^*t*×*k*1^ (Calhoun et al., 2001; Tibshirani, 2011). Here, as the successive multiplication of weight matrices leads to the larger scale of deeper dictionaries, a normalization procedure ensures reasonable performance of LASSO regression at the same scale. Subsequently, we employed the original individual signal matrix ***S***_*i*_(*i* ∈ 1, 2, …, p), along with the temporal dictionary ***D***^1^ as input to the LASSO algorithm, which produce the corresponding individual loading coefficient $\alpha_{i}^{1}$ ($\alpha_{i}^{1}$ ∈ *R*^*k*1×*n*^, *n* = 228,453). Since ***D***^1^ incorporates the group-wise temporal features, the resulting individual loading coefficients $\alpha_{i}^{1}$ obtained through regression can be considered as spatial sparse representations of each individual’s fMRI signals ***S***_*i*_ > on the common temporal dictionary ***D***^1^. Consequently, the individual loading coefficients $\alpha_{i}^{1}$ represent the individual spatial features. Here, all the loading coefficient matrix derived from LASSO regression refers to ***α***^1^ (***α***^1^ = [$\alpha_{1}^{1}$, $\alpha_{2}^{1}$, …, $\alpha_{i}^{1}$, …, $\alpha_{p}^{1}$] ∈ *R*^*k*1×(*n*×7× *p*)^, $\alpha_{i}^{1}$ = [$\alpha_{i,E}^{1}$, $\alpha_{i,G}^{1}$, $\alpha_{i,R}^{1}$, $\alpha_{i,M}^{1}$, $\alpha_{i,L}^{1}$, $\alpha_{i,S}^{1}$, $\alpha_{i,W}^{1}$] ∈ *R*^*k*1×( *n*×7)^.

Similarly, in order to derive the loading coefficient matrix $\alpha_{\text{test}}^{1}$ for testing set of each layer, the group-wise time series dictionary matrix ***D***^1^ derived from the training stage was applied to model $S_{\text{test}}^{1}$ to obtain $\alpha_{\text{test}}^{1}$ by resolving a typical l-1 regularized LASSO problem. In this work, the regularization parameter *λ*1 of LASSO regression was set as 0.1 experimentally and empirically.

### Sparse Representation Model

Although we successfully obtained individual loading coefficient matrices ***α***^1^ and $\alpha_{\text{test}}^{1}$ through LASSO regression for the training and testing sets, respectively, these features were unsuitable for classification due to their high dimensionality (***α***^1^ ∈ *R*^*k*1×*n*^, *k*1 = 128, *n* = 228,453). Therefore, our next goal was to extract the multilevel group-wise spatial patterns based on the individual spatial patterns, and finally excavate multilevel features for multitask classification that could distinguish multitask fMRI signals and reveal the distinctive organization patterns of different task stimulations. Here, we adopted a sparse representation-based model, which has already been proven as an effective algorithm in previous research to identify the intrinsic spatial functional patterns and features for multitask classification from fMRI data (Song et al., 2022; Zhang et al., 2016). Specifically, we first aggregated all the loading coefficient matrices $\alpha_{i}^{1}$ of all the subjects into one matrix ***S***^2^ for each layer of the DBN model (***S***^2^ = [$S_{1}^{2}$, $S_{2}^{2}$, …, $S_{i}^{2}$, …, $S_{p}^{2}$] ∈ *R*^*k*1×(*n*×7×*p*)^, where $S_{i}^{2}$ = [($\alpha_{i,E}^{1}$)^T^, ($\alpha_{i,G}^{1}$)^T^, ($\alpha_{i,R}^{1}$)^T^, ($\alpha_{i,M}^{1}$)^T^, ($\alpha_{i,L}^{1}$)^T^, ($\alpha_{i,S}^{1}$)^T^, ($\alpha_{i,W}^{1}$)^T^] ∈ *R*^*n*×(7× *k*1)^. Then, ***S***^2^ would be served as the input for dictionary learning and sparse representation to derive a group-wise spatial dictionary ***D***^2^ ∈ *R*^*n*×*k*2^ and the corresponding loading coefficients ***α***^2^ for each layer, respectively. Note that *k*2 represents the number of dictionary atoms, which was set as the same value as *k*1 (*k*2 = 128). Here, ***α***^2^ = [$\alpha_{1}^{2}$, $\alpha_{2}^{2}$, …, $\alpha_{i}^{2}$, …, $\alpha_{p}^{2}$] ∈ *R*^*k*2×(*k*1×7×*p*)^, where $\alpha_{i}^{2}$ = [$\alpha_{i,E}^{2}$, $\alpha_{i,G}^{2}$, $\alpha_{i,R}^{2}$, $\alpha_{i,M}^{2}$, $\alpha_{i,L}^{2}$, $\alpha_{i,S}^{2}$, $\alpha_{i,W}^{2}$] ∈ *R*^*k*2×*k*1×7^. The loss function of sparse representation model yields a sparse resolution constraint on the loading coefficient ***α***^2^ with an l1 regularization (Equation 2), where *λ*2 is a regularization parameter that can balance the regression residual and sparsity level *λ*2 was set as 0.05.

$$\mathrm{Min}\frac{1}{2}∥S^{2}-D^{2}\alpha^{2}{∥}_{F}^{2}+\lambda 2∥\alpha^{2}{∥}_{1,1}$$ (2)

To prevent ***D***^2^ from arbitrarily large values that cause the trivial solution of the optimization, the columns *d*_1_, *d*_2_, …, *d*_k_ are restricted by Equation 3.

$$C≜\{D^{2}\in R^{t\times k2},s.t.\forall j=1,\cdots ,k2,d_{j}^{T}d_{j}\leq 1\}$$ (3)

As the dictionary ***D***^2^ was obtained by a sparse representation of ***α***^1^, which comprise all individual spatial features, the learned dictionary ***D***^2^ consequently represents the group-wise spatial features. Correspondingly, $\alpha_{i}^{2}$ was a sparse representation on the common spatial dictionary ***D***^2^. Given the ability of a sparse representation model to effectively reduce the dimensionality of raw fMRI data while retaining its essential information, the resulting intrinsic features ($\alpha_{i}^{2}$) derived from the extraction of common temporal and spatial dictionaries can effectively capture the variations in spatiotemporal patterns of functional brain activity across different tasks. As a result, these intrinsic features were suitable for multitask classification.

To derive the $\alpha_{\text{test}}^{2}$ of testing set for post hoc classification analysis, we also leveraged the LASSO regression algorithm for each layer. Specifically, the loading coefficient matrix $\alpha_{\text{test}}^{1}$ was regarded as the input matrix $S_{\text{test}}^{2}$, and the dictionary matrix ***D***^2^ derived from the training stage was employed to model $S_{\text{test}}^{2}$ to learn the loading coefficient $\alpha_{\text{test}}^{2}$. All the parameters in testing stage were set the same as in the training stage.

### Parameter Selection

The determination of hyperparameters, such as the number of cross-validation folds, the number of layers and neurons of the DBN model, and the regularization parameters of the sparse representation model, was accomplished through a combination of referring to previous studies and learning from the training set, the testing set was not involved in any parameter selection process.

The choice of cross-validation folds is crucial as it offers a trade-off between precision and computational cost for performance estimation (Hansen et al., 2013). Commonly used cross-validation folds in current machine learning experiments often include twofold, fivefold, tenfold, or the leave-one-out method. In theory, while some studies suggest the tenfold or leave-one-out method may provide a higher estimated accuracy (Kohavi, 1995), some reveals that fivefold or tenfold is the optimal choice for balancing computational cost and accuracy (Hansen et al., 2013). However, due to the need for our framework to combine all individuals within the training set to extract group-wise temporal features during the training phase, the computational resource demands of the tenfold or leave-one-out method are greater. Therefore, we opted for the fivefold approach. To further validate our selection, we conducted a comparative analysis between the twofold and fivefold to assess the decoding accuracy. The findings revealed that the average decoding rate was slightly lower for the twofold compared to the fivefold, providing additional confirmation of our initial selection (Table S1).

Our selection of a four-layer, 128-neuron DBN structure was based on our previous study utilizing the neural architecture search technique (NAS) for recognizing spatiotemporal features from fMRI data (Xu, Ren, Tao, Song, & He, 2022), which effectively determined the optimal structure for DBN model with three layers and 120–150 neurons. Therefore, in our study, we defined the number of neurons as 128 and experimented with both three-layer and four-layer configurations to extract meaningful task-related temporal features. Specifically, we compared the group-wise temporal features derived from DBN model with three-layer and four-layer structures, in terms of their Pearson correlation coefficient (PCC) with task paradigm curve, based on training set (fold 5). The results revealed that the four-layer DBN outperformed in capturing temporal features, as indicated by the higher PCC values observed in the four-layer structure (Table 2). In terms of selecting the number of neurons, we took into consideration computational efficiency. We determined that selecting 128 neurons, a power of two within the desired range of 120–150, would optimize computational speed. Hence, we concluded that the optimal configuration for the DBN model with 128 neurons and four layers.

**Table 2.** Comparison of Pearson correlation coefficient for three-layer structure and four-layer structure

Structure	Layer1	Layer2	Layer3	Layer4	Mean ± *SD*
Three-layer	0.48 ± 0.12	0.52 ± 0.06	0.50 ± 0.06		0.50 ± 0.08
Four-layer	0.55 ± 0.00	0.63 ± 0.01	0.66 ± 0.03	0.71 ± 0.02	0.64 ± 0.02

The regularization parameter (*λ*) plays a crucial role in sparse representation and LASSO regression. Although no gold standard exists for determining the value of *λ*, previous studies on FBN recognition have experimentally set *λ* within the range of 0.05 to 0.5 (Ge et al., 2018; Lv et al., 2015a; Zhang et al., 2017). In our previous work on task fMRI data classification using a two-stage sparse representation approach, we conducted parameter selection experiments within the range of *λ* from 0.05 to 0.5 and found that the highest accuracy was achieved when *λ*1 = 0.1 and *λ*2 = 0.05 or 0.1 (Song et al., 2022). Here, *λ*1 and *λ*2 represent the regularization parameters for the LASSO regression and sparse representation, respectively. Therefore, in this study, we determined the *λ*1 as 0.1, and systematically changed the setting of the regularization parameter in the sparse representation *λ*2 (*λ*2 = 0.05, 0.1) while evaluating their impact on the obtained group-wise spatial features derived from training set (fold 5). The results showed that when *λ*2 was set to 0.05, a greater number of FBNs could be identified in the group-wise spatial features ***D***^2^ by comparison with the general linear model (GLM)–derived activation patterns (Table 3). Consequently, we set *λ*1 = 0.1 and *λ*2 = 0.05 as regularization parameters for LASSO regression and sparse representation stage, respectively. To further validate this, we assessed the classification accuracy on testing the dataset using these two different *λ*2 values (0.05, 0.1) while keeping *λ*1 = 0.1 for all five folds. The results demonstrated that *λ*2 = 0.05 achieved higher accuracy, reconfirming our choice (Table S2).

**Table 3.** Comparison of the number of identified FBNs cross each layer for different *λ*2 values

*λ*2	Layer1	Layer2	Layer3	Layer4
0.05	15	17	22	45
0.1	12	13	18	27

### Identification of Multilevel Temporal Patterns

As mentioned in the Deep Belief Network Model-Based Analysis section, *W*_*j*_ of the *j*-th hidden layer (*j* = 1, 2, 3, 4) represents the temporal features of group-wise tfMRI for respective layer (Figure 1B). Here we used PCC as a metric to identify the task-related temporal features (Benesty, Chen, Huang, & Cohen, 2009; Lv et al., 2015a). Specifically, we first calculated the task paradigm curves convolved with hemodynamic response function (HRF). Next, we computed the PCC values between the convolved task paradigm curves and the atoms in the group-wise temporal features ***D***^1^ derived from the DBN model, following standard procedures employed in previous studies (Kay, Rokem, Winawer, Dougherty, & Wandell, 2013; O’Reilly, Woolrich, Behrens, Smith, & Johansen-Berg, 2012). The PCC of the identified temporal features and the task-based stimulus can be defined as Equation 4.

$${\text{P}}_{\text{corr},\text{c}}=\text{corr}(D_{c}^{1},\text{TASK})$$ (4)

Here, $D_{c}^{1}$ refers to the c-th component in temporal features ***D***^1^ derived from DBN stage (c = 1, ⋯, *k*1). TASK represents the task paradigm curves convolved with HRF. Essentially, P_corr,c_, measures the temporal similarity between the temporal patterns of $D_{c}^{1}$ and the task stimulus. The atoms with the highest PCC value in group-wise temporal features ***D***^1^ were chosen to represent the multilayer temporal features.

### Identification of Multilevel Spatial Patterns

The multilevel spatial patterns can also be identified in the second stage of sparse representation model. Specifically, the $S_{i,t}^{1}$ can be factorized into ***D***^1^ and the loading coefficient $\alpha_{i,t}^{1}$, which represent the group-wise temporal features and the individual spatial features, respectively. Here, *i* refers to *i*-th subjects (*i* ∈ 1, 2, …, p, and p = 48 in this work), *t* means *t* kind of task, *t* ∈ Φ = {*E*, *G*, *R*, *M*, *L*, *S*, *W*}. To further derive the group-wise spatial features, the transposition of ***α***^1^ could be then decomposed into ***D***^2^ and ***α***^2^ as shown in Equation 5. Since the transpose of $\alpha_{i,t}^{1}$ can be expressed as dictionary ***D***^2^ multiplied by loading coefficient $\alpha_{i,t}^{2}$ (Equation 5), the relationship between $S_{i,t}^{1}$ and ***D***^1^, ***D***^2^, ***α***^2^ can be deduced as Equation 6 shown, which also consistent with previous studies (Liu et al., 2017a; Song et al., 2022).

$$S_{i,t}^{2}={\left(\alpha_{i,y}^{1}\right)}^{T}=D^{2}\times \alpha_{i,t}^{2}$$ (5)

$$S_{i,t}^{1}=D^{1}\times \alpha_{i,t}^{1}=D^{1}\times {\left(D^{2}\times \alpha_{i,t}^{2}\right)}^{T}$$ (6)

Since all subjects share the same group-wise temporal dictionary ***D***^1^, the common dictionary ***D***^2^ contained group-wise spatial patterns, of which atoms could be used to define the FBNs. Thus, the corresponding multilayer spatial features were derived from the common dictionary ***D***^2^ for each layer of the proposed framework (the fourth and fifth panels in Figure 1B).

We then identified the spatial correlation coefficient (SCC) to quantify the similarity between spatial patterns obtained from the proposed framework and the GLM-derived activation patterns. Specifically, the GLM-based analysis was performed individually, followed by group-wise analysis using FSL FEAT (https://www.fmrib.ox.ac.uk/fsl/feat5/index.html). The group-level GLM-based results were employed for comparison. More details of GLM analysis are available in previous literature (Lv et al., 2015b). The SCC is defined in Equation 7 (Harrison et al., 2008; Zuo et al., 2010):

$$R\left(X,T\right)=\frac{\sum_{p=1}^{n}\left(X_{p}-\bar{X}\right)\left(T_{p}-\bar{T}\right)}{\sqrt{\sum_{p=1}^{n}{\left(X_{p}-\bar{X}\right)}^{2}\cdot \sum_{p=1}^{n}{\left(T_{p}-\bar{T}\right)}^{2}}}$$ (7)

where ***X*** is the spatial functional network derived by the proposed framework, ***T*** represents the GLM-derived activation template, and *n* refers to the number of voxels of whole brain.

### SVM-Based Classification Method

To further classify multitask fMRI signals, we performed fivefold cross-validation to evaluate the classification performance of the proposed framework. As the linear SVM has optimization and generalization capability in limited sample sizes, as well as its proven effectiveness in multiclass classification (Chang & Lin, 2011; Jang et al., 2017), we conducted multitask classification analysis based on linear SVM classifier, which was established by the LIBSVM toolbox (Chang & Lin, 2011). For each layer, as the loading coefficient ***α***^2^ contains both temporal and spatial features embedded in fMRI signals, we first trained the SVM classifier using ***α***^2^ derived from training set, and then evaluated the classification performance by feeding the $\alpha_{\text{test}}^{2}$ of testing set into the trained SVM model. Based on the true label of seven tasks for each loading coefficient $\alpha_{\text{test}}^{2}$, the classification accuracy of each layer in each fold was defined as the percentage of correctly predicted samples. The final classification accuracy for each layer is the average of five folds for seven tasks. We then calculated the specificity of each fold for each layer, and the final specificity for each layer is the average of the five folds.

### ROA-Based Analysis

The further goal aimed at uncovering discriminative functional components for multitask classification. Inspired by the successful use of the ratio-of-activation (ROA) in identifying discriminative components for decoding resting state fMRI (rsfMRI) and tfMRI (Zhang et al., 2016), we raised a novel ROA metric to identify the key components for seven-task classification. The ROA of the *j*-th row in loading coefficients ***α***^2^ could be defined as follows:

$$\begin{array}{l} N_{t}={\left|\alpha^{2}\left(j,k\right)\right|}_{0},kth\;\text{column}\;\text{belongs}\;\text{to}\;\text{task}\left(t\right)\\ {\text{ROA}}_{j}=\sqrt{\frac{1}{T}\sum_{t=1}^{T}{\left(N_{t}-\bar{N_{t}}\right)}^{2}} \end{array}$$ (8)

In Equation 8, ***α***^2^ represent all the individual spatiotemporal features, ***α***^2^ = [$\alpha_{1}^{2}$, $\alpha_{2}^{2}$, …, $\alpha_{i}^{2}$, …, $\alpha_{p}^{2}$] ∈ *R*^*k*2×(*k*1×7×*p*)^ (*k*1 = *k*2 = 128, *p* = 48); *i* refers to *i*-th subject (*i* ∈ 1, 2, …, *p*). *t* represents task index (*t* ∈ 1, 2, …, 7), and *T* represents the number of task paradigms (i.e., 7 in our work). Task (*t*) represents each of the seven different tasks. *N*_*t*_ represents the activation level for each task, and $\bar{N_{t}}$ represents the average of *N*_*t*_ (*t* = 1, ⋯, 7). Here, the activation level *N*_*t*_ was defined by counting the number of nonzero entries marked as each task in the corresponding each row vector of ***α***^2^ (*t* ∈ 1, 2, …, 7). As ***α***^2^ is a sparse matrix, the task with a higher count of nonzero elements in the row vectors of ***α***^2^ is deemed to be more “active.” Therefore, *N*_*t*_ represents each task’s activation level in the row vectors of ***α***^2^. The ROA was calculated by counting the standard deviation of *N*_*t*_ across the seven tasks. A larger ROA value (i.e., larger standard deviation) indicates greater differences in activity levels across the seven tfMRI signals, which were more discriminative for multitask classification.

To validate that the components of higher ROA values capture greater capacity in classifying the multitask fMRI signals, an experiment was designed as illustrated below. After sorting the ROA values for all components (i.e., rows in loading coefficients ***α***^2^) from highest to lowest, we iteratively adopted more rows sorted by their ROA values in ***α***^2^ as feature inputs for training the SVM classifier, that is, the components with higher ROA values were used preferentially for training. Afterward, the corresponding components of $\alpha_{\text{test}}^{2}$ from testing set were entered into the trained SVM model to evaluate the classification accuracy. Specifically, to define the key components with greater capacity for multitask classification in each layer, we have repeated this ROA analysis using ***α***^2^ derived from each layer of proposed model. Here we applied the same classification scheme described in the previous section, SVM-Based Classification Method.

After establishing the ROA metric for the classification features ***α***^2^, our subsequent objective is to elucidate the neural implications of these classification features. Given that each row of ***α***^2^ corresponds to each column of ***D***^2^ (i.e., each atom in ***D***^2^), and these atoms can be mapped back to brain space, we thus established a relationship between the brain activations derived from the atoms in ***D***^2^ and the ROA values of the row vectors of ***α***^2^. This connection allows us to interpret neural implications of classification features.

## RESULTS

### Classification Performance of Multitask fMRI Signals

**Figure 2.**
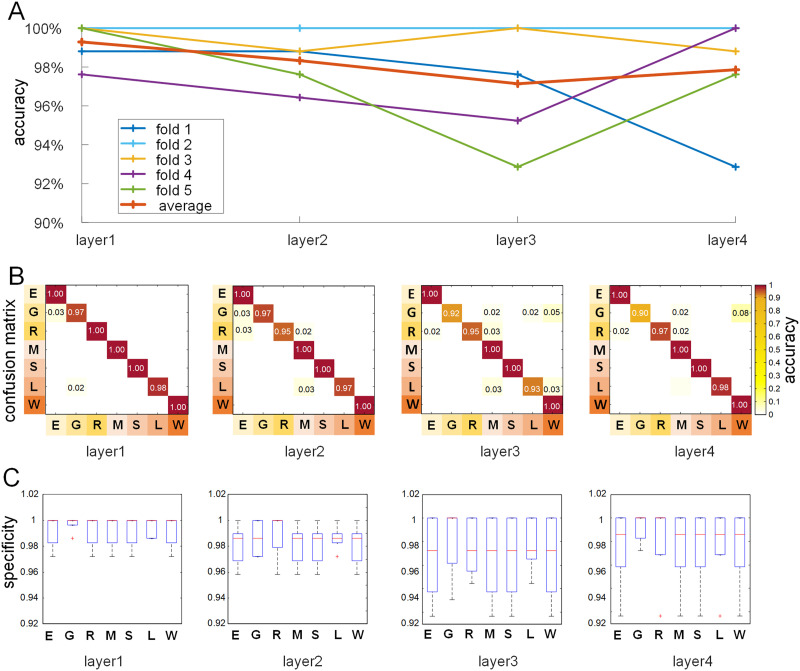
Classification performance. (A) The classification accuracy of fivefold in each layer. (B) The average confusion matrices of fivefold cross-validation on the seven tasks. (C) The average specificity of fivefold cross-validation classification on the seven tasks.

By applying the proposed DBN-SR framework to multitask fMRI data using fivefold cross-validation strategy, our results reveal that the fMRI data of seven tasks can be accurately classified. In detail, the classification accuracy for fivefold ranges from 92.86% to 100%, with an average accuracy of 97.86% ± 3.42% (mean ± *SD*) in the layer 4 (Figure 2A), which demonstrated the proposed framework can effectively uncover the inherent differences in composition patterns of multitask fMRI signals.

We also explored the classification performance based on features derived from each layer of the proposed framework (Figure 2). The trend of the classification accuracy curves for five folds is relatively steady, with an average accuracy of 98.15% ± 0.90% (mean ± *SD*) (Figure 2A). Moreover, the average accuracies across fivefold from layer 1 to layer 4 are 99.29%, 98.33%, 97.14%, and 97.86%, respectively. We depicted confusion matrices for each layer to represent the average classification accuracy of the seven tasks, as shown in Figure 2B. The results indicate that all the average classification accuracies for seven tasks across fivefold are greater than 95% in each layer, except for three major confusions, that is, gambling task in layer 3 and layer 4, relational task in layer 2 and layer 3, and language task in layer 3 (Figure 2B). In addition, the specificity of classification results of the first two layers is slightly higher than that of the deeper two layers (Figure 2C). Overall, the classification performance of the shallower layers is relatively better than that of the deeper layers.

### Identified Multilevel Temporal and Spatial Patterns of Multitask fMRI Signals

#### Multilevel temporal patterns.

**Figure 3.**
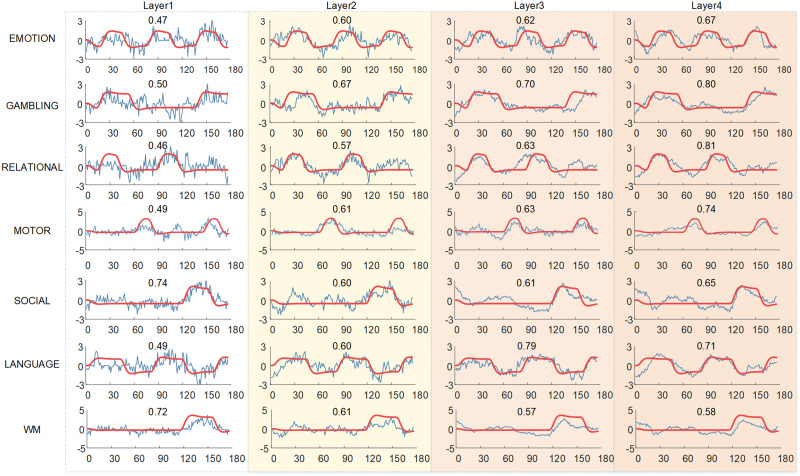
Comparison of group-wise temporal patterns for seven tasks across different layers, including the identified temporal features (blue lines) and the task paradigms (red lines). The quantitative similarities (PCC) of identified temporal features with task paradigms are also provided. The *y*-axis represents the stimulus response amplitude, while the *x*-axis represents time point. The background colors represent different layers of our DBN-SR model. The lighter colors represent shallower layers, while the darker colors represent deeper layers.

Our DBN-SR-based framework can effectively identify the temporal patterns of multitask fMRI signals at multiscale (Figure 3). In each layer, we quantitatively compared the PCC of the identified temporal features and each task-based stimulus. Those atoms with the highest PCC value in temporal dictionary ***D***^1^ were chosen to represent the task-related temporal patterns. We randomly select one training fold as an example to show the representative temporal patterns for each layer (fold 5) (Figure 3). The average PCC values of seven tasks for all fivefold can be found in Supporting Information Table S6.

The overall multilevel temporal patterns are relatively consistent with the task design paradigms. Specifically, the average PCC of seven tasks from Layer1 to Layer4 is 0.55 ± 0.12, 0.61 ± 0.03, 0.65 ± 0.07, and 0.71 ± 0.08 (mean ± *SD*), respectively, where the highest correlation is observed in Layer4 (Figure 3). Intriguingly, there exists a gradient in the resolution of temporal patterns derived from different layers. In the shallow layer, all the identified temporal patterns are mixed with many random noises, resulting in a relatively poor correlation with task paradigms. In comparison, in the deeper layer, the temporal patterns are smoother and more consistent with the original task design curves, indicating that the DBN-SR model can filter noises in each layer while keeping useful information of brain activities, which agrees with the former research (Huang et al., 2018; Zhang et al., 2020).

#### Multilevel spatial patterns.

**Figure 4.**
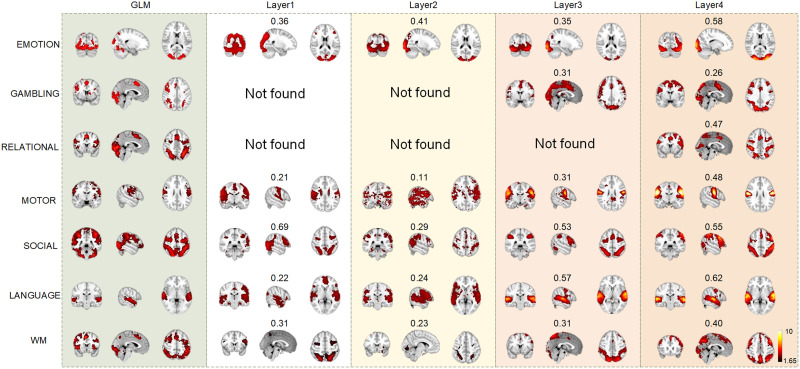
Comparison of group-wise spatial patterns for seven tasks across different layers. The spatial correlation coefficient (SCC) between each identified spatial pattern and GLM-derived activation pattern is labeled on top of each brain map.

Our framework can also effectively identify the spatial patterns from different layers. The most predominant spatial patterns identified by the proposed framework are the task-evoked FBNs, including emotion, gambling, relational, motor, social, language, and working memory. In each layer, we quantitatively compared the SCC of the identified spatial patterns and the GLM-derived activation patterns. Those atoms with the highest SCC value in spatial dictionaries ***D***^2^ were chosen to represent the spatial pattern. We randomly selected one training fold to illustrate the representative FBNs for each layer (Figure 4).

Overall, the spatial patterns are generally consistent with the GLM-derived activation patterns, with increasingly precise resolution from shallow to deep layers. Quantitatively, the average SCC of seven tasks from layer1 to layer4 is 0.36 ± 0.20, 0.26 ± 0.11, 0.40 ± 0.12, and 0.48 ± 0.12 (mean ± *SD*), respectively, where the highest SCC is observed in layer4 (Figure 4). Intriguingly, there exist distinct differences among spatial patterns derived from different layers. The spatial patterns across layers show a trend of increasing consistency with the GLM-derived activation patterns, and are more compact in deeper layers for most tasks. Meanwhile, more FBNs can be found in the deeper layers compared with shallow layer. For example, some FBNs cannot be identified in the first three layers, such as FBNs related to gambling and relational tasks (Figure 4).

**Figure 5.**
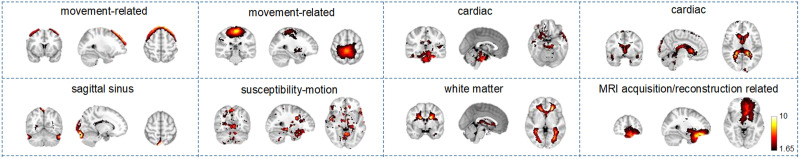
Identified artifact components, including movement-related, cardiac-related, sagittal sinus, susceptibility-motion, white-matter, and MRI acquisition/reconstruction-related.

Apart from FBNs, the proposed framework can also effectively detect various artifact-related components. Specifically, the atoms in spatial dictionary ***D***^2^ can represent the group-wise spatial features and can be mapped back to the 3D brain volume. Subsequently, we manually inspected whether spatial map matched the known types of artifacts based on previous study (Salimi-Khorshidi et al., 2014). Through this process, we found several artifact-related components, including movement-related, cardiac-related, sagittal sinus, susceptibility-motion, white-matter, and MRI acquisition/reconstruction-related (Figure 5).

Overall, our effective DBN-SR model is capable of characterizing the multilevel spatiotemporal features of brain function. The quantitative analysis further demonstrates that, in deeper layer, the representative temporal features correspond well with task design curves, and the spatial features are relatively more consistent with the GLM-derived activation. In addition to task-evoked functional components, our framework could also effectively identify artifact components from group-wise multitask fMRI data, laying the groundwork for further research into the functional role of these components in multitask classification.

### Identification of Discriminative Features by ROA Analysis

**Figure 6.**
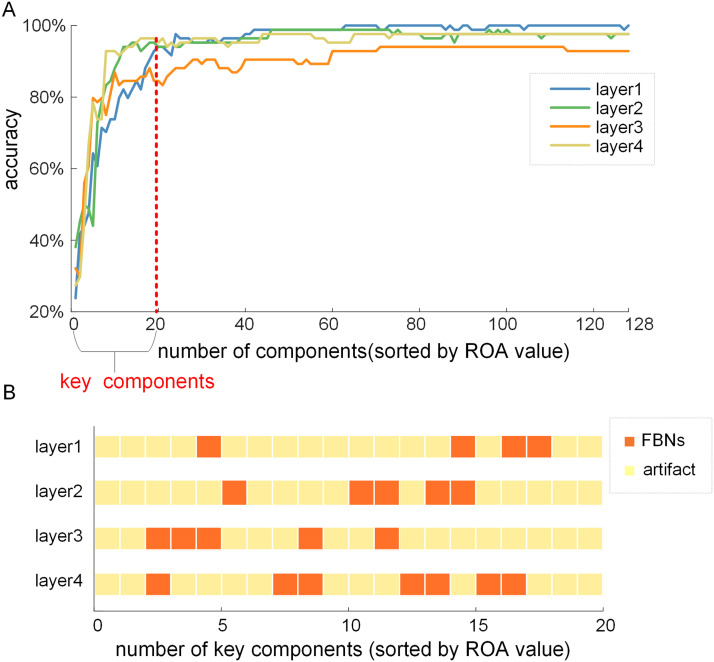
ROA classification results in each layer (fold 5). (A) Classification accuracy for SVM-based classification of four layers using the different number of components sorted by their ROA values. (B) The composition of 20 key components sorted by ROA value across each layer.

As depicted in the ROA-Based Analysis section, we first computed the ROA index by sorting the ROA values of all the components in loading coefficients ***α***^2^ of the training set, then, in order to evaluate the classification performance, the corresponding components in the loading coefficient $\alpha_{\text{test}}^{2}$ of testing set were fed sequentially into the trained SVM classifier according to the ROA index. Here, the classification results of each layer on one randomly selected testing fold dataset (fold 5) using different number of components, sorted by their ROA values, are illustrated in Figure 6A. While the number of components increases from 1 to 20, the accuracy curves of four layers grow monotonically, and the average accuracy of all curves rises to 91.96%. When more than 20 components are included for classification, the accuracy curves of four layers exhibit a plateau with accuracies reaching close to 100%, indicating that the additional components with lower ROA values contribute less to the successful classification of multitask signals. Thus, the top 20 components with higher ROA values can be regarded as key components for the classification task to some extent. Generally, our method can effectively disclose the key components with great classification capacity. In addition, the findings are consistent across different testing folds, hence the additional results of the other four folds are included in the Supporting Information (Figures 2S–5S).

To further investigate the neural implications of key components with greater classification capacity, we inspected the spatial patterns of the top 20 key components identified by ROA analysis in each layer. By further analyzing the composition of the 20 key components in each layer, we found that these key atoms are either FBNs or artifact-related components, which were identified by visually examining their spatial patterns with established templates and further calculating their SCC with GLM-derived activation maps.

Intriguingly, our results show that the top 20 key components in the four layers are largely composed of artifacts, while the proportion of FBNs in key components is small as a whole. On the other hand, the proportion of FBNs is relatively higher in deeper layers compared to shallower layers (Figure 6B). This conclusion aligns with the findings when using the top 40 components as key components (Figure S8).

## DISCUSSION

In this study, we proposed a hybrid spatiotemporal deep belief network and sparse representation framework to decode multitask fMRI signals on a relatively small cohort dataset. Our framework could classify fMRI signals of seven tasks with high accuracy and detect multilevel temporal patterns and FBNs, suggesting the effectiveness of the proposed method. In addition, our framework can reveal key components including artifact components and FBNs in multitask classification and uncover their underlying neurological implication.

Our proposed framework is composed of several elements, including the DBN model, LASSO regression, sparse representation, and SVM classifier, resulting in a relatively complex structure. Nevertheless, our framework achieved a relatively higher classification accuracy in comparison to prior research that also conducted classification of seven task states on the HCP dataset (Huang, Xiao, & Wu, 2021; Wang et al., 2020), while also yielding interpretable classification components. Specifically, Wang et al. (2020) reported two standard machine learning algorithms, namely MVPA-SVM and DNN, and Huang et al. (2021) proposed a novel framework (CRNN) incorporating multiple modules such as CNN, recurrent neural network, and attention mechanism. The average accuracy of our framework (98.15%) is much higher than that of MVPA-SVM (69.2%) and comparable to the accuracies of the DNN-based model (93.7%) and CRNN-based model (94.31%) (Huang et al., 2021; Wang et al., 2020). Additionally, the neuroscientific implications of their results remain elusive. In conclusion, our proposed model achieved higher decoding accuracy than these models, while also providing a more comprehensive and interpretable methodology for decoding fMRI data.

Furthermore, our model unveils multilevel temporal and spatial patterns, demonstrating a resolution gradient spanning from shallow to deep layers. Specifically, in the deeper layers, the identified temporal features are better correlated to the original task paradigm curves. Meanwhile, more diverse FBNs can be detected, and the spatial features show more consistency with the GLM-derived activation patterns in deeper layers.

Intriguingly, although more higher order FBNs can be detected in deeper layers, the classification accuracy using features for multitask classification derived from deeper layers is lower than that of shallower layers, indicating that these higher order FBNs are not very helpful for multitask classification. To validate this observation, we specifically selected only FBN components from all available components across all five folds for multitask classification, resulting in an average accuracy of 97.08% ± 2.14% (mean ± *SD*), slightly lower than the classification rate obtained using all components (98.15% ± 0.90%) (Table S3). The possible reason is that the FBNs evoked by different cognitive tasks may have coactivated brain regions, thus the FBN components alone may not fully reveal the potential fundamental differences in functional composition patterns of multitask fMRI data. On the other hand, ROA-based analyses indicate that artifact components occupy higher proportion of key components for multitask classification in shallower layers than that in deeper layers, along with higher classification accuracy and specificity in the shallower layers. These findings suggest that the artifact components play an important role in multitask fMRI signal classification, which is also consistent with previous research, where the artifact components of the EEG signal are significantly more informative than brain activity concerning classification accuracy (McDermott et al., 2022).

While our study provides novel insight into the core functional components in decoding multitask fMRI signals, it is important to note that there are three limitations. The first limitation is the manual setting of parameters for the DBN and sparse representation framework, mainly including the number of neuron nodes and layers in the DBN and the sparsity penalty parameter of SR. Thus, automatic optimization of model parameters is one of the future research directions. The second limitation stems from our inability to detect FBNs related to gambling and relational tasks within the first two to three layers of the DBN-SR framework. This could be attributed to more noise present in the group-wise temporal features ***D***^1^ extracted at lower levels (Figure 1). Additionally, LASSO regression may not be well-suited for handling noisy shallow features, thus making it challenging for LASSO regression to accurately capture the underlying spatial patterns. To address this limitation, future studies could explore alternative regression approaches that are better suited for handling noisy shallow features, thereby improving the accurate acquisition of the underlying spatial patterns. The third limitation is that our study employed a relatively small dataset, consisting of 60 individuals out of 68 from HCP Q1 dataset. To assess the robustness of our model, we included the remaining eight individuals from the same dataset as a hold-out dataset, six of which do not have complete data for all seven tasks (Table S4). However, this does not affect their suitability as an independent lock box dataset to test the performance of our trained model. The results revealed that the average decoding accuracy for these eight individuals (96.43%) was comparable to the fivefold cross-validation accuracy of the 60 individuals (Table S5), suggesting the robustness of our model. Nonetheless, we acknowledge that a larger dataset would lend further support to our findings. In future work, we aim to apply our model to more extensive or multicenter datasets to evaluate its generalizability and robustness.

Overall, with the superiority of interpretability and effectiveness of the DBN-SR model on small datasets, our framework could potentially be useful to differentiate abnormal brain function in clinical research.

## SUPPORTING INFORMATION

Supporting information for this article is available at https://doi.org/10.1162/netn_a_00334.

## AUTHOR CONTRIBUTIONS

Limei Song: Conceptualization; Investigation; Methodology; Writing – original draft. Yudan Ren: Conceptualization; Funding acquisition; Resources; Supervision; Validation; Writing – review & editing. Shuhan Xu: Data curation; Formal analysis. Yuqing Hou: Funding acquisition; Supervision; Validation. Xiaowei He: Funding acquisition; Supervision; Validation.

## FUNDING INFORMATION

Yudan Ren, National Natural Science Foundation of China (https://dx.doi.org/10.13039/501100001809), Award ID: 62006187. Yudan Ren, Youth Innovation Team Foundation of Education Department of Shaanxi Province Government, Award ID: 21JP119. Yudan Ren, China Postdoctoral Science Foundation Funded Project, Award ID: 2021M702650. Xiaowei He, National Natural Science Foundation of China (https://dx.doi.org/10.13039/501100001809), Award ID: 61971350. Xiaowei He, National Natural Science Foundation of China (https://dx.doi.org/10.13039/501100001809), Award ID: 12271434. Yuqing Hou, Key Research and Development Program Project of Shaanxi Province, Award ID: 2020SF-036. Xiaowei He, Natural Science Basic Research Program of Shaanxi, Award ID: 2023-JC-JQ-57.

## TECHNICAL TERMS

Support vector machine (SVM): A machine learning algorithm that finds the optimal hyperplane to separate data into different classes.
Multitask classification: Process of using machine learning algorithms to classify multiple task-based fMRI data simultaneously.
Deep belief network (DBN): A type of deep learning network composed of multiple layers of hidden units, used for feature extraction and classification.
Least absolute shrinkage and selection operator (LASSO): A regularization technique that encourages sparse solutions by adding a penalty term to the loss function.
Sparse representation (SR): A method of representing data using a small number of nonzero coefficients or features.
Cross-validation: A technique in machine learning that assesses model performance by splitting data into subsets for training and testing.
Regularization parameters: Values in machine learning that prevent overfitting by adding a penalty to the loss function.
Resolution gradient: A gradual change in the level of detail or specificity of features or patterns, from shallow to deep layers in a neural network.
