# Supplementary material for: A hybrid spatiotemporal deep belief network and sparse representation-based framework reveals multilevel core functional components in decoding multitask fMRI signals
Source: Netw Neurosci. 2023 Dec 22;7(4):1513–32. doi: 10.1162/netn_a_00334 (PMC10745082; doi:10.1162/netn_a_00334)
Supplement: Supplementary file 1 [file netn-7-4-1513-s001.pdf]

Supplementary materials

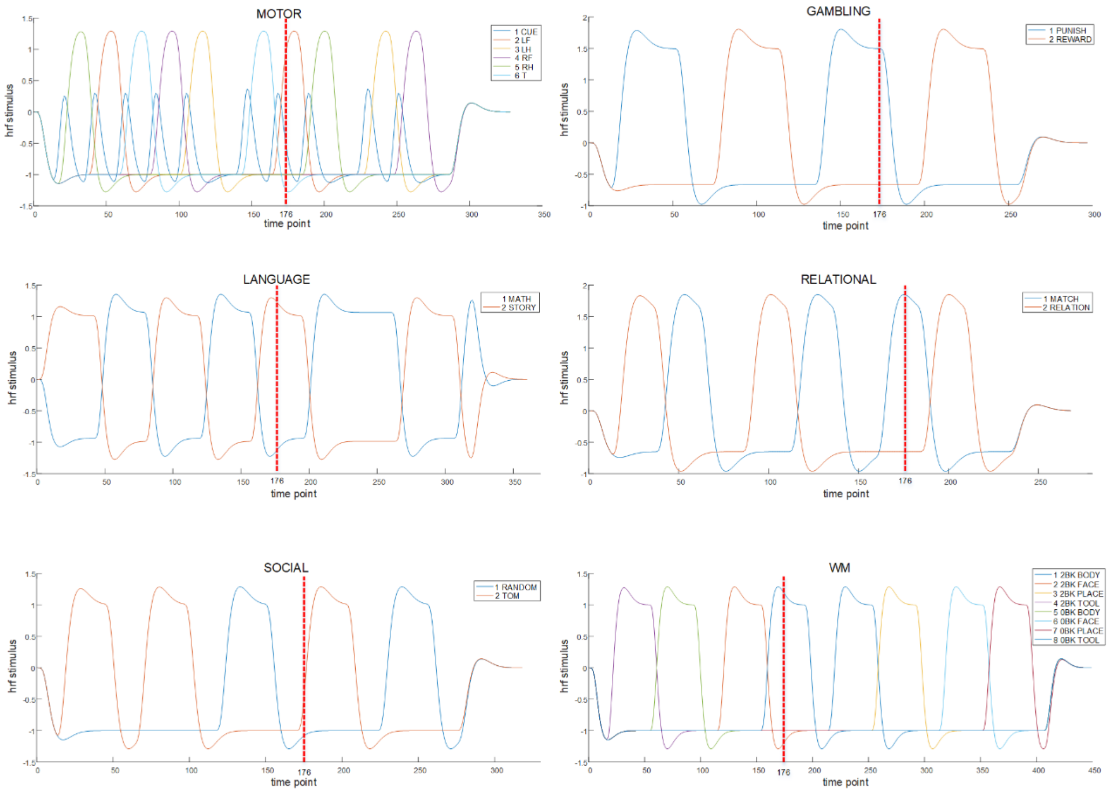

Supplemental Figure 1. Truncation of six task designs

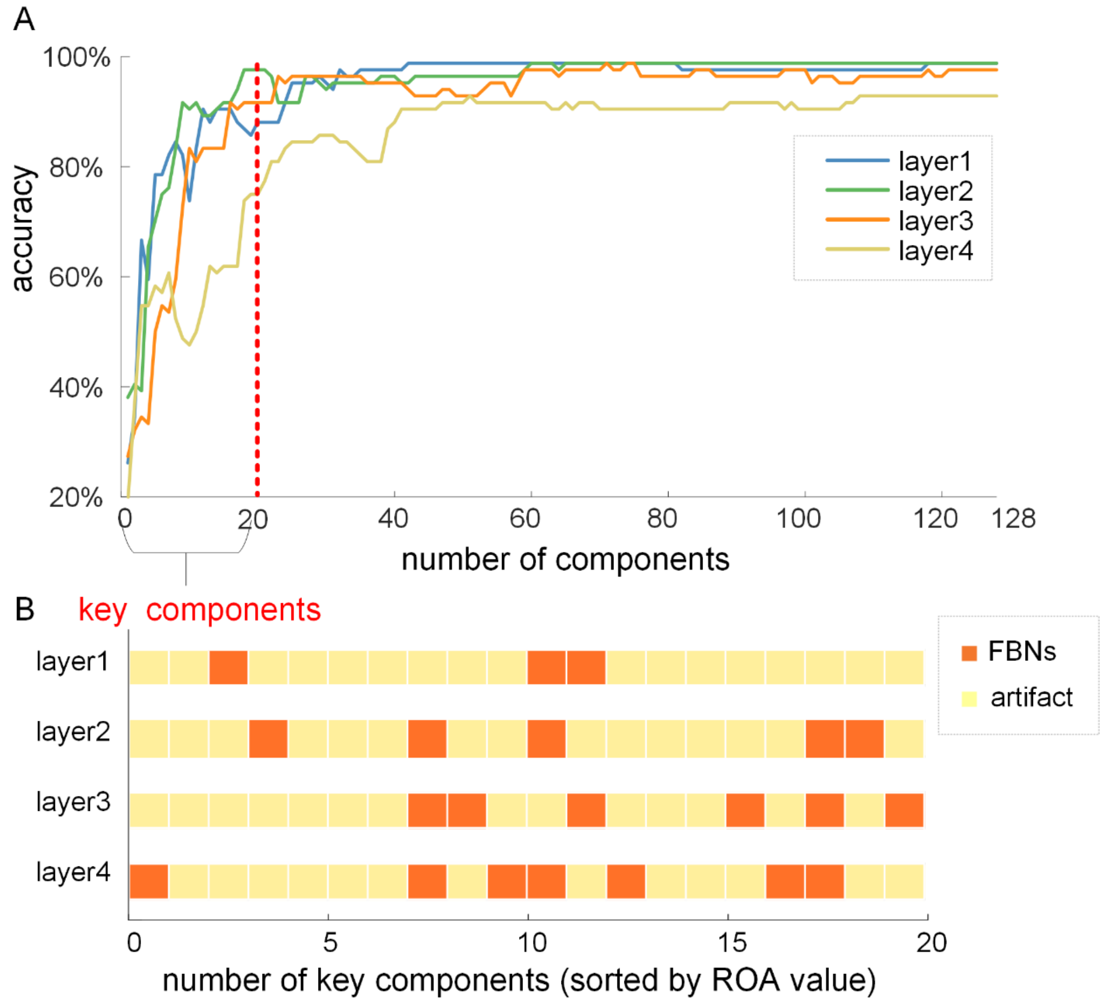

Supplemental Figure 2. Classification performance (fold1). (A) The classification accuracy of five-fold in each layer. (B) The average confusion matrices of five-fold cross-validation on the seven tasks. (C) The average specificity of five-fold cross-validation classification on the seven tasks.

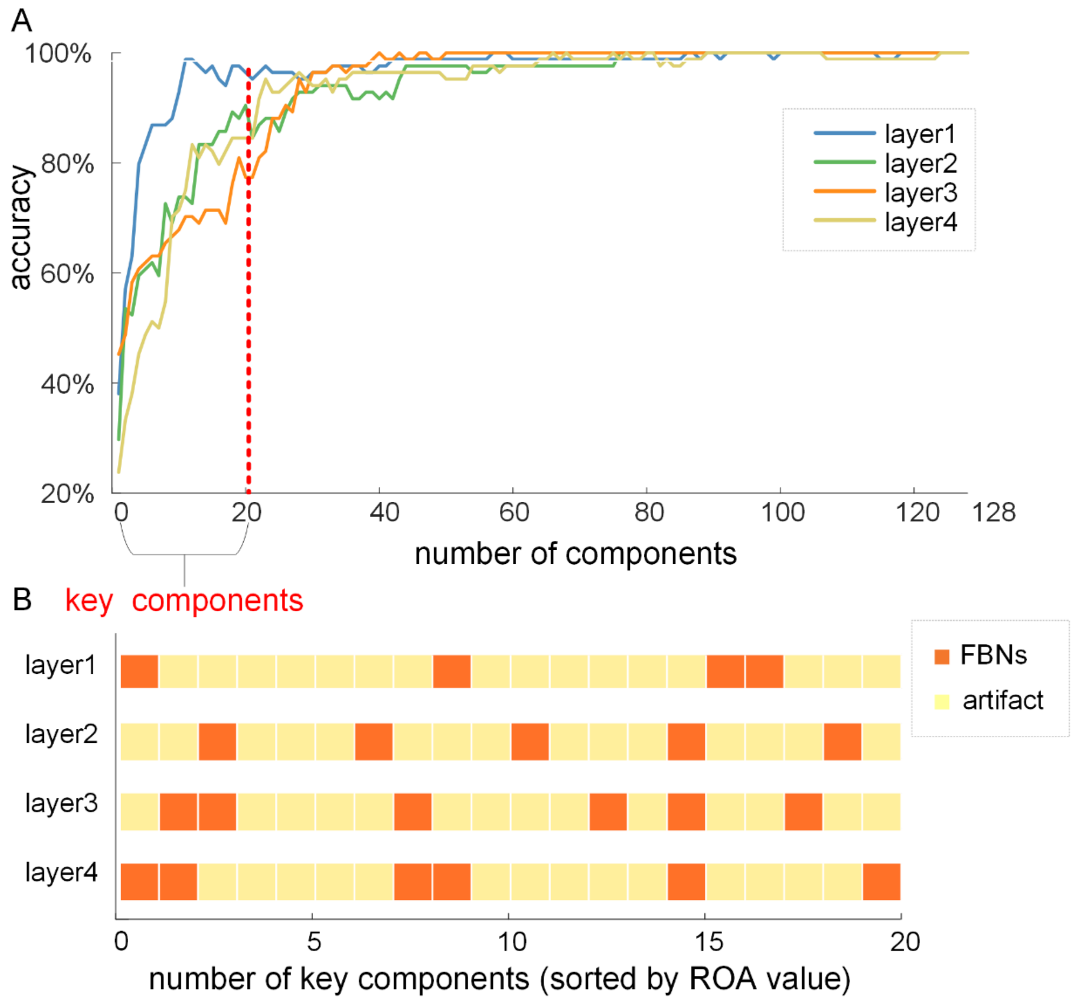

Supplemental Figure 3. Classification performance (fold2). (A) The classification accuracy of five-fold in each layer. (B) The average confusion matrices of five-fold cross-validation on the seven tasks. (C) The average specificity of five-fold cross-validation classification on the seven tasks.

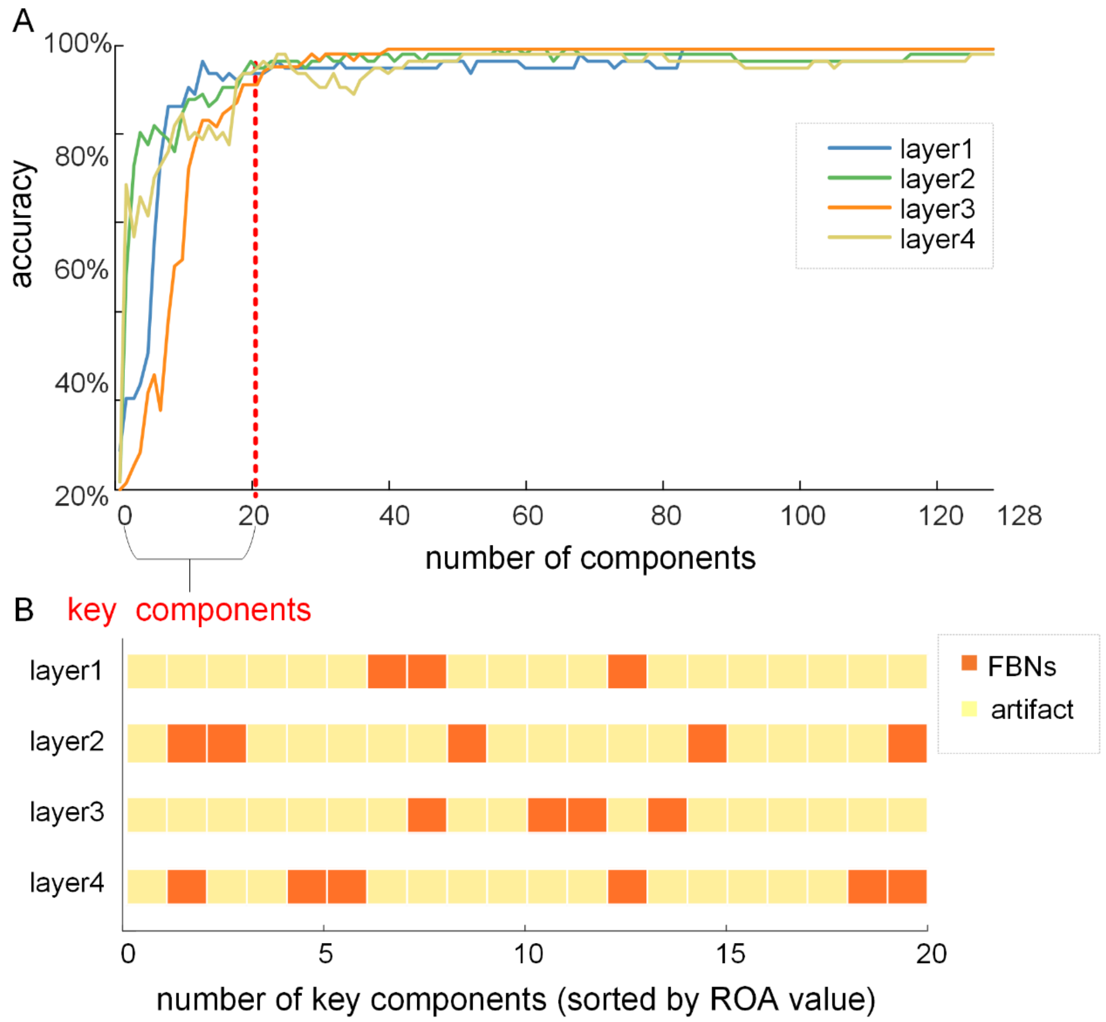

Supplemental Figure 4. Classification performance (fold3). (A) The classification accuracy of five-fold in each layer. (B) The average confusion matrices of five-fold cross-validation on the seven tasks. (C) The average specificity of five-fold cross-validation classification on the seven tasks.

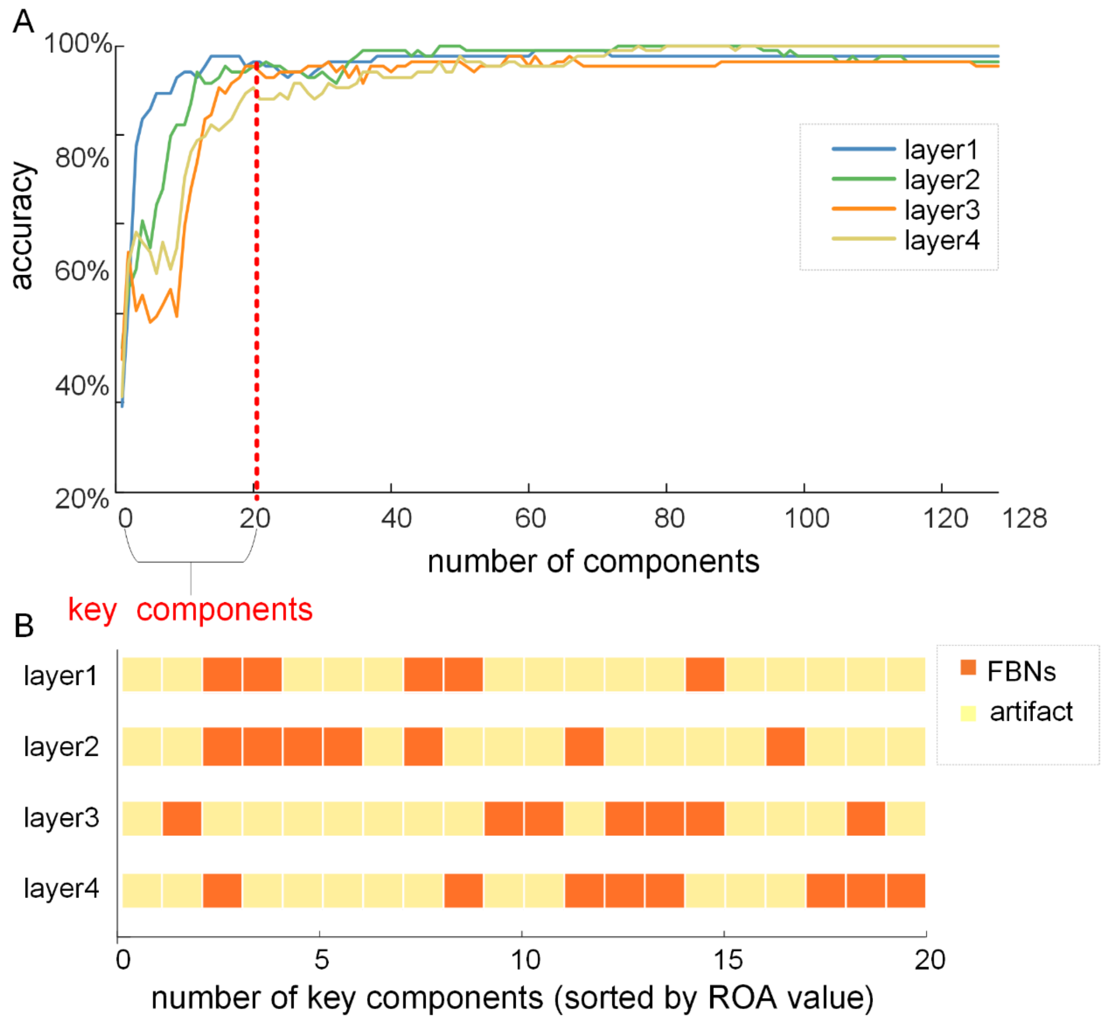

Supplemental Figure 5. Classification performance (fold4). (A) The classification accuracy of five-fold in each layer. (B) The average confusion matrices of five-fold cross-validation on the seven tasks. (C) The average specificity of five-fold cross-validation classification on the seven tasks.

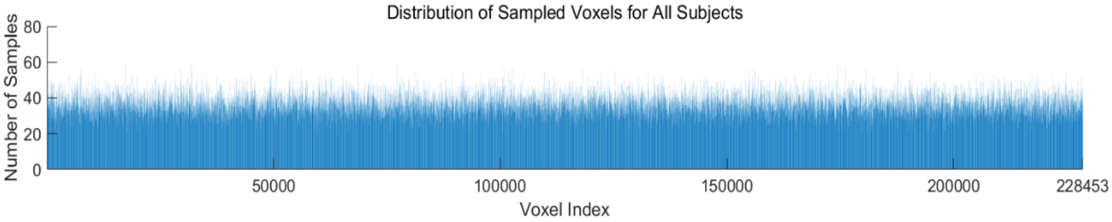

Supplemental Figure 6. Distribution of 10% randomly selected voxels across all subjects.

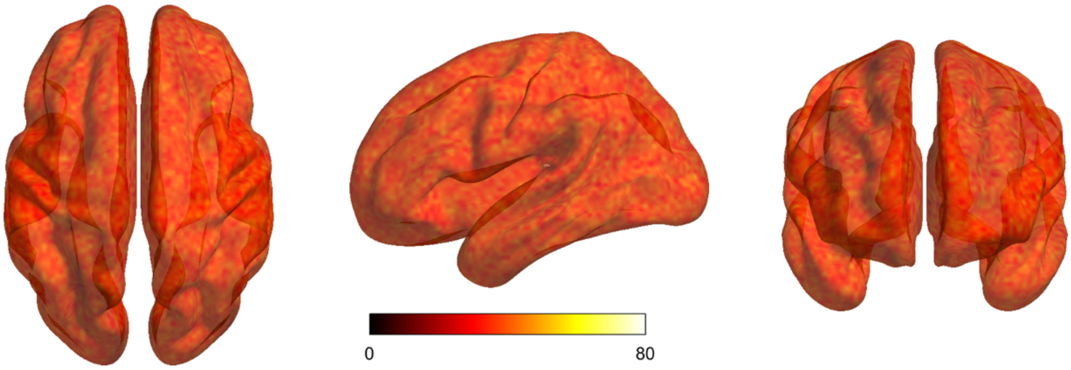

Supplemental Figure 7. Spatial distribution of randomly sampled 10% voxels across all subjects.

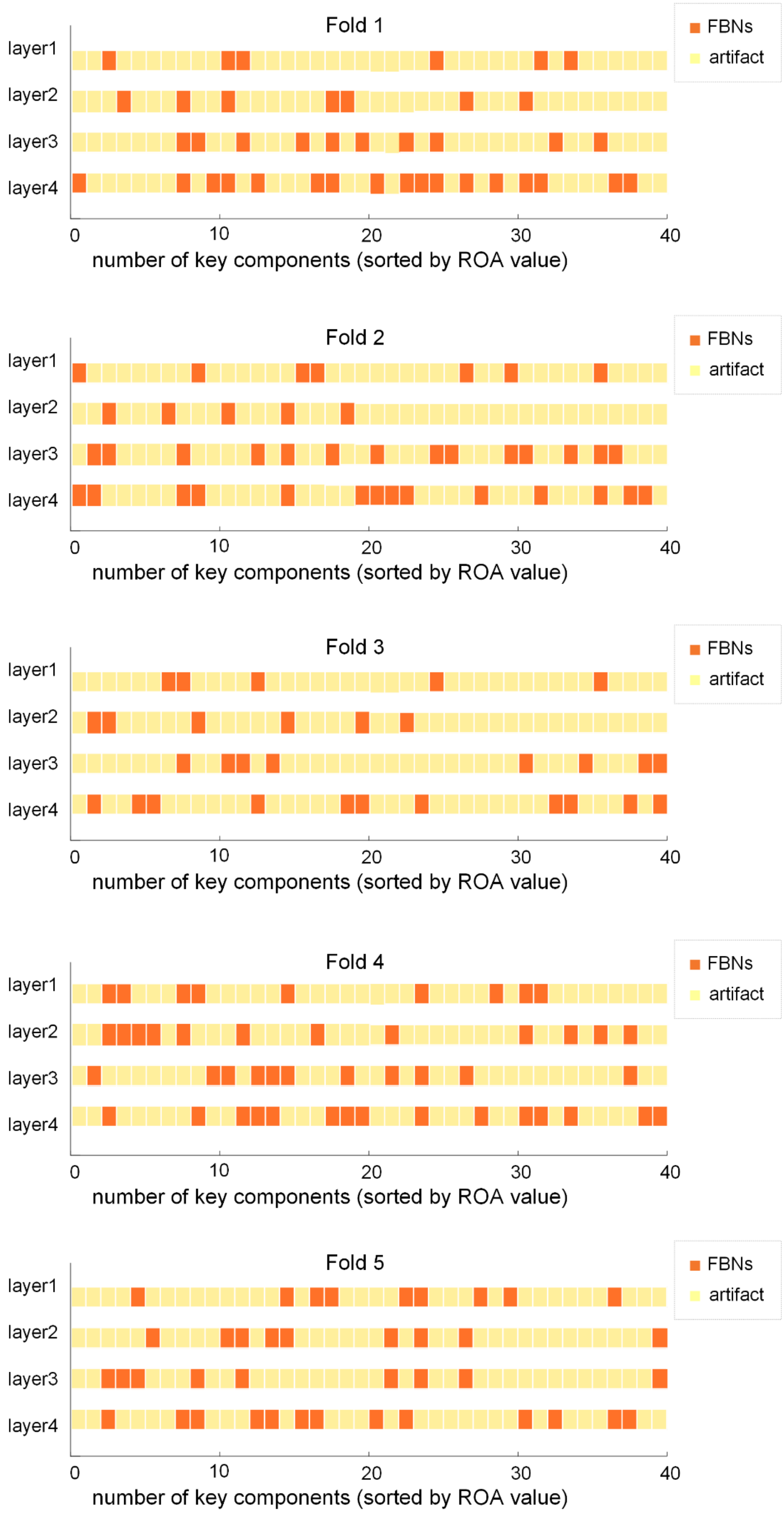
Fold 1
Fold 2
Fold 3
Fold 4
Fold 5
FBNs
artifact
layer1
layer2
layer3
layer4
0
10
20
30
40
number of key components (sorted by ROA value)

Supplemental Figure 8. The composition of the top 40 components sorted by ROA across each layer for 5-fold.

Supplemental Table 1 Comparison of average classification accuracies for 2-fold and 5-fold (Mean±SD).

	Layer 1	Layer 2	Layer 3	Layer 4	Mean±SD
2-fold	99.52%±0.00%	98.57%±1.34%	98.57%±1.34%	90.48%±6.06%	96.79%±4.23%
5-fold	99.29%±2.83%	98.33%±0.00%	97.14%±0.69%	97.86%±2.03%	98.15%±0.90%

Supplemental Table 2 Average Decoding Accuracy for 5-Fold Cross Validation and 4 Layers with Varying λ2 Values (Mean±SD).

λ1\λ2	0.1	0.05
0.1	92.74%±6.60%	98.15%±1.43%

Supplemental Table 3. Comparison of accuracy using network components only and all components.

Components	Layer1	Layer2	Layer3	Layer4	Mean±SD
All	99.29%	98.33%	97.14%	97.86%	98.15%±0.90%
Network-only	99.76%	97.14%	94.52%	96.90%	97.08%±2.14%

Supplemental Table 4. Task data of additional 8 subjects in lock box

Sub\Task	EMOTION	GAMBLING	RELATIONAL	MOTOR	SOCIAL	LANGUAGE	WM
1	not include	not include					
2							
3			not include				
4							
5			not include				
6		not include					
7			not include				
8						not include	

Supplemental Table 5. Comparison of decoding rates for 8 subjects (49 Samples) in the lock box and 5-fold average decoding rates for 60 subjects.

Layer	Lock box (8 subjects)	5-fold average (60 subjects)
1	100%	99.29%
2	91.84%	98.33%
3	95.91%	97.14%
4	97.96%	97.86%
Mean±SD	96.43%±3.48%	98.15%±0.90%

Supplemental Table 6 Comparison of the average PCC of each task for each layer across

5-fold.

	Layer1	Layer2	Layer3	Layer4	Mean±SD
Fold1	0.55±0.13	0.63±0.07	0.64±0.06	0.69±0.08	0.63±0.06
Fold2	0.55±0.12	0.63±0.05	0.7±0.05	0.75±0.05	0.66±0.09
Fold3	0.55±0.12	0.64±0.05	0.64±0.06	0.69±0.06	0.63±0.06
Fold4	0.55±0.12	0.62±0.03	0.69±0.05	0.69±0.05	0.64±0.07
Fold5	0.55±0.12	0.61±0.03	0.65±0.07	0.71±0.08	0.63±0.07
